# A rare case report of multiple myeloma presenting with paralytic ileus and type II respiratory failure due to hypercalcemic crisis

**DOI:** 10.1097/MD.0000000000009215

**Published:** 2017-12-29

**Authors:** Yuchen Guo, Liang He, Yiming Liu, Xueyuan Cao

**Affiliations:** a Department of Gastrointestinal Surgery, First Hospital of Jilin University; b Department of Pharmacy, Second Hospital of Jilin University, Changchun, China.

**Keywords:** hypercalcemia, multiple myeloma, paralytic ileus

## Abstract

**Rationale:**

Paralytic ileus is characterized by the signs and symptoms of intestinal obstruction but without any mechanical lesions in the intestinal lumen. Several medical and surgical conditions can lead to this ailment, such as electrolyte disturbances that impair intestinal motility. However, hypercalcemia secondary to multiple myeloma as a major cause of paralytic ileus has rarely been reported.

**Patient concerns:**

The patient got severe constipation with difficulty in the passage of both gas and feces for 7 days.

**Diagnoses:**

The patient was diagnosed with a small intestinal obstruction initially and then developed type II respiratory failure. Investigations revealed hypercalcemic crisis, and examination of a bone marrow aspirate was consistent with multiple myeloma.

**Interventions:**

Conservative treatment was administered for the intestinal obstruction, consisting of food and water deprivation, gastrointestinal decompression, colonic irrigation, intravenous fluid transfusion, anti-inflammatory therapy. Invasive respiratory support was provided after type II respiratory failure occurred and salmon calcitonin was used to reduce the blood calcium level. Further therapy was given by the Department of Hematology and Oncology in our hospital after the diagnosis of multiple myeloma.

**Outcomes:**

Spontaneous respiration and gastrointestinal function were restored after the correction of hypercalcemia.

**Lessons:**

An appropriate diagnostic approach is needed in emergency practice to identify the paralytic ileus and type II respiratory failure caused by hypercalcemia secondary to multiple myeloma.

## 1 Introduction

Intestinal obstruction is one of the most common causes of acute abdominal pain. Nevertheless, the condition can be difficult to diagnose because it can be caused by a diverse array of conditions, including postoperative intra-abdominal adhesions, superior mesenteric vessel thrombosis, electrolyte disturbances, and even occult diseases.^[1,2]^ Here, we report a case in which intestinal pseudo-obstruction (paralytic ileus) as well as type II respiratory failure developed because of hypercalcemic crisis in a patient with multiple myeloma.

## 2 Case report

**Figure 1:**
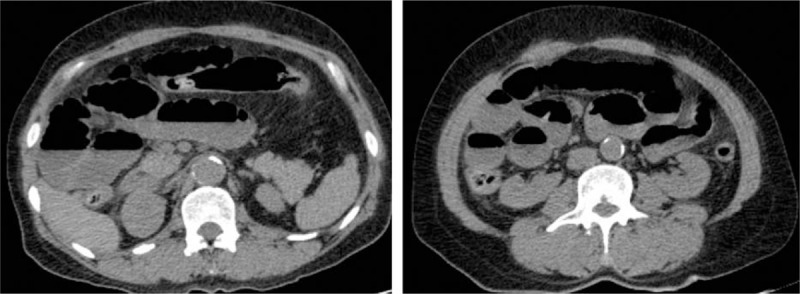
Computed tomography scan reveals small intestinal obstruction. No obvious mechanical obstruction is found on CT scanning. The small intestine is generally dilated, and air–fluid levels can be seen in the intestine. CT = computed tomography

A 47-year-old woman was admitted to our hospital because of severe constipation with difficulty in the passage of both gas and feces for 7 days. Approximately 3 weeks before admission into our hospital, she had been diagnosed with renal inadequacy at a local hospital. Blood tests at that hospital revealed a serum creatinine (SCr) level of 238 μmol/L (normal range, 40–106 μmol/L) and a blood urea nitrogen (BUN) level of 14.6 μmol/L (normal range, 1.7–8.3 μmol/L). During the course of medical treatment for her renal condition, she developed ileus. Upon presenting to our hospital, she reported no history of abdominal surgery. An examination revealed mild tenderness in the epigastrium on palpation, with slightly increased muscle tone and no rebound tenderness. Abdominal auscultation showed decreased bowel sounds (about 3 per minute). An abdominal computed tomography scan displayed small intestinal obstruction (Fig. 1). Laboratory tests revealed the following: SCr, 255.4 μmol/L (normal range, 46–92 μmol/L); BUN, 18.53 μmol/L (2.5–6.1 μmol/L); blood calcium, 3.49 μmol/L (2.10–2.55 μmol/L); white cell count, 9.34 × 10^9^/L (3.5 × 10^9^–9.5 × 10^9^/L); neutrophil count, 6.97 × 10^9^/L (1.8 × 10^9^–6.3 × 10^9^/L; 75% of all white cells); hemoglobin, 98 g/L (115–150 g/L); and platelet count, 272 × 10^9^/L (125 × 10^9^–350 × 10^9^/L). Owing to the increased blood calcium level, hyperparathyroidism was suspected; however, the parathyroid hormone (PTH) level was found to be normal (PTH, 14 pg/mL; reference range, 12–88 pg/mL).

**Figure 2:**
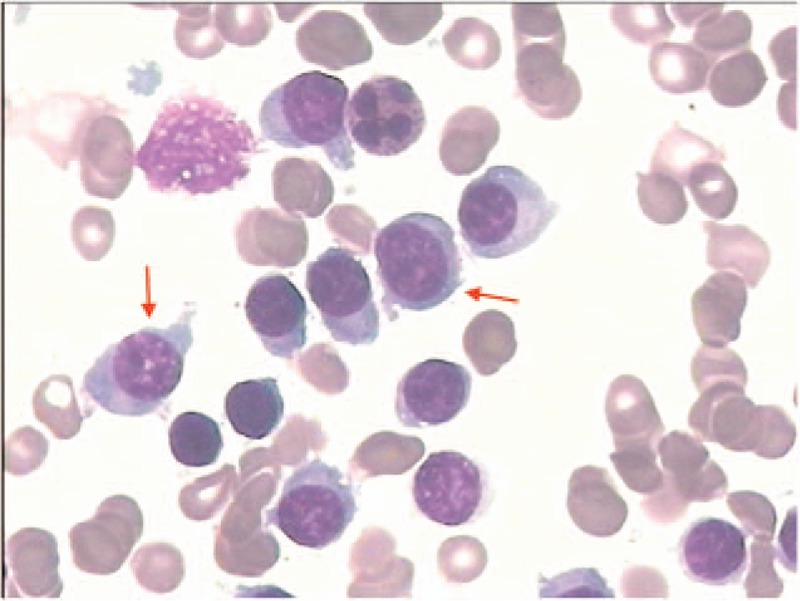
Bone marrow needle biopsy confirms a diagnosis of multiple myeloma. The oncocytes are immature and poorly differentiated plasmocytes. Vacuole can be seen in the nuclei of the oncocytes, and slight chromatin loosening is present.

**Figure 3:**
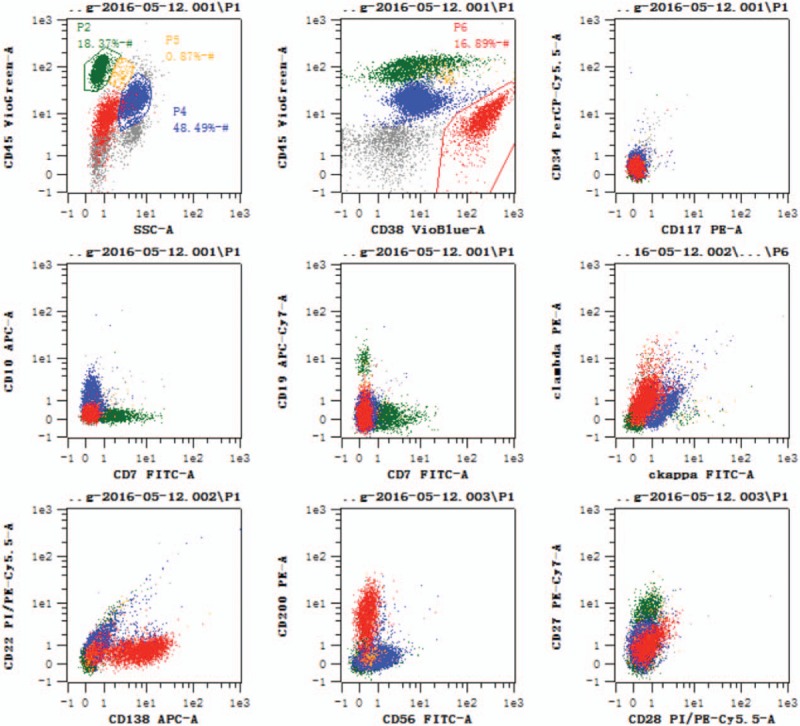
Flow cytometric immunophenotyping of bone marrow indicates that P6 cells (expressing CD38, CD138, CXCR4, CD200, and cLambda^dim^ and not CD7, CD117, CD56, CD34, CD19, CD20, CD10, CD22, CD27, CD28, CD13, CD33, and cκ) make up 16.89% of karyocytes; that is, 16.89% of the sample cells are abnormal monoclonal plasma cells. APC = allophycocyanin, CD = cluster of differentiation, CT = computed tomography, Cy = cyanine, FITC-A = fluorescein isothiocyanate - area, PE-A = phycoerythrin - area, PI = propidium iodide, SSC = side scatter.

The patient was diagnosed with a small intestinal obstruction, renal inadequacy, hypercalcemia, and mild anemia. Conservative treatment was administered for the intestinal obstruction, consisting of food and water deprivation, gastrointestinal decompression, colonic irrigation, intravenous fluid transfusion, anti-inflammatory therapy, and octreotide Sandostatin (1ml:1mg, provided by Novartis Pharma Stein AG, Switzerland). Although receiving this conservative therapy, the patient developed type II respiratory failure. At that time, her blood calcium level was 3.70 μmol/L. Invasive respiratory support was provided, and salmon calcitonin (1ml:50IU, provided by Novartis Pharma Stein AG, Switzerland) was used to reduce the blood calcium level. Additionally, we performed tests for inorganic phosphorus, serous free-λ light chains, B2-microglobulin, and 24-hour urinary λ-light chains and κ-light chains to explore the etiopathogenesis of the hypercalcemia. The results (and normal ranges) were as follows: 24-hour urinary λ-light chains, 1035.00 mg/24 h (<7.8 mg/24 h); serous free-λ light chains, >1230 mg/L (8.30–27.00 mg/L); and B2-microglobulin, 20.00 mg/L (0.7–1.8 mg/L). From these results, multiple myeloma was suspected. Therefore, a bone marrow needle biopsy was performed, and the results confirmed a diagnosis of multiple myeloma (Figs. 2 and 3). After 3 days of intravenous fluid transfusion and salmon calcitonin therapy, the patient's blood calcium level decreased to 2.72 μmol/L, and spontaneous respiration and gastrointestinal function were restored. She also was able to pass gas and feces, and resume her usual diet. She was then transferred to the Department of Hematology and Oncology in our hospital to receive therapy for multiple myeloma, and the subsequent treatment was not under our care.

## 3 Discussion

In our patient, multiple myeloma presented as renal dysfunction, paralytic ileus, and ultimately type II respiratory failure because of hypercalcemic crisis. Hypercalcemia is an uncommon cause of small bowel obstruction. The most common causes of hypercalcemia are primary hyperparathyroidism and malignancy.^[3,4]^ Multiple myeloma is a malignancy of the plasma cells; the condition is initially localized to the bone marrow, from where cells eventually disseminate and form bone lesions.^[5]^ Multiple myeloma accounts for approximately 10% of all hematological malignancies.^[6]^ Hypercalcemia, which can range in presentation from mild to severe and life threatening, is the most common metabolic complication of multiple myeloma and occurs in approximately one-third of patients.^[7]^ The primary cause of hypercalcemia in myeloma is widespread tumor-induced bone destruction. This is primarily because of increased osteoclastic bone resorption induced by potent cytokines expressed or locally secreted by the myeloma cells or overexpressed by other cells in the local microenvironment.^[8]^ This bone resorption in turn leads to the efflux of calcium into the extracellular fluid, leading to hypercalcemia.

The clinical presentation of hypercalcemia is frequently dependent on the blood calcium level. With a blood calcium level of 3 mmol/L or less, patients may be asymptomatic. At levels of 3 to 4 mmol/L, patients may present with symptoms such as dry mouth, anorexia, vomiting, polyuria, polydipsia, depression, abdominal pain, constipation, or confusion. With a calcium level of 4 mmol/L or greater, a life-threatening coma, known as hypercalcemic crisis, may develop.^[7]^ Most studies emphasize that it is associated with rapid deterioration of the central nervous system as well as cardiac, renal, and gastrointestinal dysfunction, resulting in renal failure, encephalopathy, cardiac dysrhythmias, and even death.^[3]^

Hypercalcemia can also lead to paralytic ileus. Tsai et al^[9]^ reported a case of acute colonic pseudo-obstruction secondary to hypercalcemia. Cases of paralytic ileus caused by hypercalcemic crisis because of adult T-cell leukemia^[10]^ or because of chemotherapy for multiple myeloma have also been reported.^[11–13]^ However, few cases of hypercalcemic crisis secondary to multiple myeloma itself and presenting with paralytic ileus have been reported. In this case, we believe hypercalcemia played a crucial role in the pseudo-obstruction of the intestine, because no definite mechanical cause for the ileus was found in our patient. Moreover, the occurrence of ileus was accompanied by a rise in serum calcium levels, and correction of hypercalcemia rapidly relieved the paralytic ileus and restored gastrointestinal motility. Furthermore, no other electrolyte disturbance was found during the course of ileus. Additionally, type II respiratory failure occurred when the blood calcium level reached 3.70 μmol/L. We considered that the respiratory failure was attributable to respiratory muscle paralysis or pulmonary calciphylaxis secondary to hypercalcemia.^[14]^

## 4 Conclusion

Hypercalcemia secondary to multiple myeloma may create conditions that lead to paralytic ileus and type II respiratory failure, and thus, a diagnostic approach that can identify this condition is needed in emergency practice.
